# Magnetic Isotropy/Anisotropy in Layered Metal Phosphorous Trichalcogenide MPS_3_ (M = Mn, Fe)Single Crystals

**DOI:** 10.3390/mi9060292

**Published:** 2018-06-11

**Authors:** Zia ur Rehman, Zahir Muhammad, Oyawale Adetunji Moses, Wen Zhu, Chuanqiang Wu, Qun He, Muhammad Habib, Li Song

**Affiliations:** 1 National Synchrotron Radiation Laboratory, CAS Center for Excellence in Nanoscience, University of Science and Technology of China, Hefei 230029, China; zurehman@mail.ustc.edu.cn (Z.u.R.); zahir@mail.ustc.edu.cn (Z.M.); oyawaletj@mail.ustc.edu.cn (O.A.M.); sa2016@mail.ustc.edu.cn (W.Z.); wucq@mail.ustc.edu.cn (C.W.); hqun@mail.ustc.edu.cn (Q.H.); mhabib@mail.ustc.edu.cn (M.H.); 2 Department of Applied Physics, University of Karachi, Karachi-75270, Pakistan

**Keywords:** layered material, bulk single crystal, isotropy, anisotropy, chemical vapor transport (CVT), density-functional theory (DFT)

## Abstract

Despite the fact that two-dimensional layered magnetic materials hold immense potential applications in the field of spintronic devices, tunable magnetism is still a challenge due to the lack of controllable synthesis. Herein, high-quality single crystals MPS_3_ (M= Mn, Fe) of millimeter size were synthesized through the chemical vapor transport method. After systemic structural characterizations, magnetic properties were studied on the bulk MPS_3_ layers through experiments, along with first principle theoretical calculations. The susceptibilities as well as the EPR results evidently revealed unique isotropic and anisotropic behavior in MnPS_3_ and FePS_3_ crystals, respectively. It is worth noting that both of these materials show antiferromagnetic states at measured temperatures. The estimated antiferromagnetic transition temperature is 78 K for bulk MnPS_3_ and 123 K for FePS_3_ crystals. The spin polarized density functional theory calculations confirmed that the band gap of the antiferromagnetic states could be generated owing to asymmetric response all over the energy range. The ferromagnetic state in MnPS_3_ and FePS_3_ is less stable as compared to the antiferromagnetic state, resulting in antiferromagnetic behavior. Additionally, frequency-dependent dielectric functions for parallel and perpendicular electric field component vectors, along with the absorption properties of MPS_3_, are thoroughly investigated.

## 1. Introduction

Magnetism has been proven to be widely helpful in the understanding of the quantum nature of materials. Although 2D transition metal dichalcogenides (TMDs) have been the center of attention in the recent era, transition metal chalcogenophosphates (TMCs) with general formula of MPX3 (M = Mn, Fe, Ni, Co and X = S, Se) have also proven to be unique materials with their low-dimensional magnetic properties. These MPX3 have secured renewed interest owing to their importance, not only for fundamental research, but also as potential candidates for numerous technological applications [1,2,3,4,5,6,7,8,9,10]. They belong to one of the few layered systems having 2D lattice for both magnetic and crystallographic systems. These materials possess two spin channels, one conducting and the other insulating. This family of materials is considered to be the best suited for applications in spintronic [11,12]. Over the last few years, MPX3 have gained attention because of their enormous potential as a material for next generation electronics. In this regard, MnPS_3_ has been the focus because of electron valley freedom coupled with antiferromagnetic order [13]. A minute amount of magnetic field leak makes these antiferromagnetic materials a well-suited choice for use in robust data storage applications [14,15]. In recent years, layered antiferromagnetic materials have been used to induce deterministic switching without an external magnetic field [16]. Additionally, the higher magnetic resonance frequency of antiferromagnetic materials, in comparison with ferromagnetic materials, has attracted their use in providing high-speed data processing [17].

MnPS_3_ and FePS_3_ belong to the same MPX3 class of materials with isotropic and anisotropic magnetic susceptibilities, respectively [18]. Both MnPS_3_ and FePS_3_ are layered magnetic materials with weak Van der Waal forces separating the layers. The presence of the Van der Waal gap rules out the possibility of superexchange pathways as well as minimizes the possibility of direct exchange owing to metal-metal interlayer distance of ~6.5 Å and 6.4 Å in MnPS_3_ and FePS_3_, respectively. These materials can be exfoliated into a single layer [19] and possess a magnetic ground state, dependent on transition metal element [18].

MPX3 materials have been mostly investigated for their intercalation properties [9]. These are wide bandgap, highly resistive semiconductors having bandgap of the order of 3 eV. Bandgaps for MnPS_3_ and FePS_3_ are approximately 2.4 and 1.5 eV, respectively. The adequate amount of bandgap makes these materials a suitable semiconductor for optoelectronic [20], optoelectronic material processing [6], while MnPS_3_ has also been realized as a promising photocatalyst for water splitting [21]. MnPS_3_ is optically transparent with a greenish color [22], while FePS_3_ has a brownish appearance. More importantly, the magnetic character of MnPS_3_ arises from Mn^2+^ having spin 5/2 that forms a honeycomb lattice in a, b plane. The weak Van der Waal interlayer coupling is attributed to S atoms, but the antiferromagnetic phase transition at 78 K points towards an interplane exchange that can be associated with some degree of metal-ligand covalency [23,24]. FePS_3_ is a striking compound because of the fact that it exhibits stronger magnetic interactions between Fe^2+^ ions in comparison with Mn^2+^ as well as due to the reason that electronic structure of the Fe^2+^ ions demonstrate strong anisotropy [18,25]. The isotropic/anisotropic nature affects the frequency-dependent dielectric and absorption properties of MPS_3_ as well. All these facts make these MPS_3_ ideal candidates for 2D magnetic as well as optoelectronic systems.

Herein, we report the chemical vapor transport (CVT) synthesis of highly crystalline, millimeter-size MPS_3_ (M = Mn, Fe) single crystals. Furthermore, the magnetic isotropic/anisotropic properties of bulk MPS_3_ single crystals were tested using electron paramagnetic resonance (EPR) and vibrating sample magnetometer (VSM). Experimental results revealed the antiferromagnetic nature of these materials with MnPS_3_ exhibiting isotropic whereas, FePS_3_ showed highly anisotropic properties measured by magnetic susceptibilities. The magnetic and dielectric properties of these materials were confirmed using first principle spin polarized density-functional theory (DFT) calculations.

## 2. Experiments

The millimeter-sized MnPS_3_ and FePS_3_ single crystals were prepared using CVT method. The charge for MnPS_3_ and FePS_3_ was prepared by using a stoichiometric amount of Mn, P, S and Fe, P, S for MnPS_3_ and FePS_3_, respectively. Iodine was used as transport agent for this growth. The charge was kept in a 17-cm long silica tube, and was sealed under vacuum (≈$10^{-1}$ atm). The sealed ampoule was placed inside a two-zone furnace with a hot zone temperature of 650 °C and cold zone temperature of 600 °C for 7 days. The reaction yielded pale green colored single crystals and brownish single crystals at the low temperature end of ampoule for MnPS_3_ and FePS_3_, respectively.

The structural study of as-prepared samples was carried out by using X-ray diffraction (XRD) and high-resolution transmission electron by microscope (TEM); phonon spectrum was studied by using Raman Spectroscopy, chemical composition by using X-ray photoelectron spectroscopy (XPS), the morphology of the samples was investigated by using scanning electron microscope (SEM). The magnetic properties of these bulk crystal samples were studied by using electron paramagnetic resonance (EPR), and physical properties measurement system (PPMS) through vibrating sample magnetometer (VSM).

## 3. Results and Discussions

**Figure 1:**
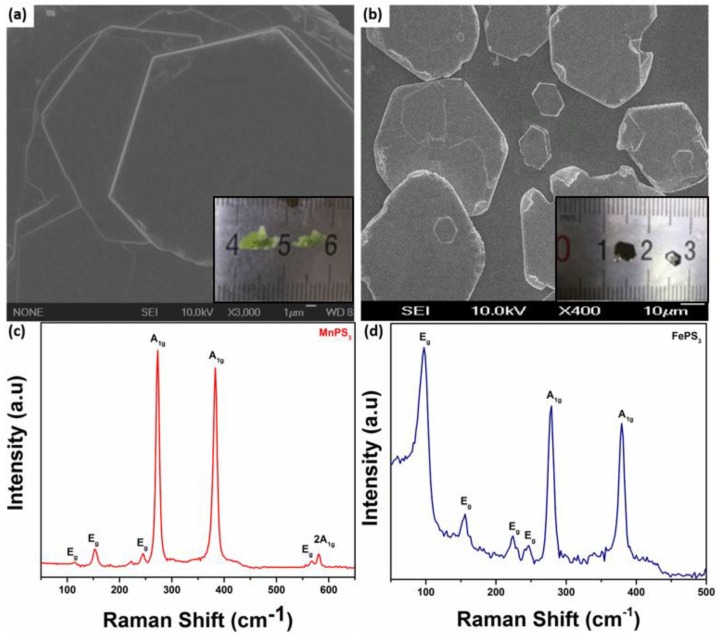
(**a**,**b**) Scanning electron microscope (SEM) images, inset shows millimeter sized as grown single crystals and (**c**,**d**) Raman spectra of as-obtained MnPS_3_ and FePS_3_ single crystals, respectively.

Typical SEM images of as-obtained MnPS_3_ and FePS_3_ samples are shown in Figure 1a,b. It can be seen that both MnPS_3_ and FePS_3_ exhibit smooth surfaces with roughly hexagonal morphology. The SEM images also show a layered structure of MPS_3_. Inset Figure 1a,b depict millimeter size as grown single crystals of MnPS_3_ and FePS_3_, respectively. Figure 1c,d represent the Raman spectra of MnPS_3_ and FePS_3_ recorded at room temperature using 532 nm laser excitation. For MnPS_3_, the Raman peaks at 114.3, 152.2, 245 and 568 cm^−1^ are identified as E_g_ vibrating mode, and 273.3, 283.1 and 580 cm^−1^ are recognized as A_1g_ mode [21]. In the case of FePS_3_, the Raman peaks at 97.3, 155.9, 224.1, 246.8 cm^−1^ are identified as E_g_ vibrating mode whereas, peaks at 279 and 379.2 cm^−1^ are referred to as A_1g_ mode [23].

**Figure 2:**
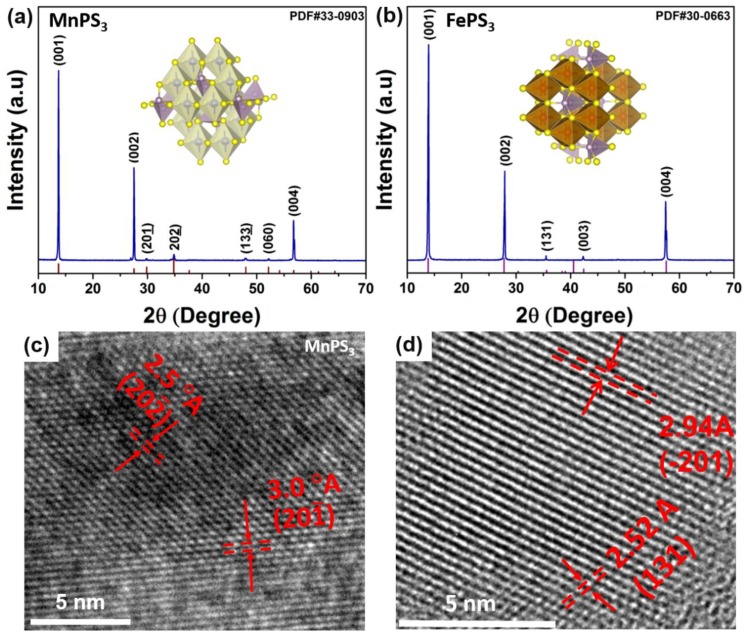
(**a**,**b**) X-ray diffraction patterns and (**c**,**d**) high resolution transmission electron by microscope (TEM) images for MnPS_3_ and FePS_3_ single crystals prepared by ball-milling and sonication process.

The X-ray diffraction data (XRD) of the samples were analyzed to study the crystal planes and crystallinity of MnPS_3_ and FePS_3_ single crystals. It is evident from Figure 2a,b that these results are well in accordance with the standard JPCDS cards belonging to (PDF#33-0903, PDF#30-0663) MnPS_3_ and FePS_3_, respectively. The crystal lattice and phase identification revealed that both MnPS_3_ and FePS_3_ exhibit high-quality single crystals with major (001) and (002) peaks. The absence of any impurity peak suggests that these samples have pure phases. Inset in Figure 2a,b shows the crystallographic model of MnPS_3_ and FePS_3_ along (001) plane, respectively. Figure 2c,d displays the TEM images of MnPS_3_ and FePS_3_, suggesting that both samples are highly crystalline with growth as single crystal. The d-spacing calculated from Figure 2c,d is estimated as 0.25 nm and 0.30 nm corresponding to (20$\bar{2}$) and (20$\bar{1}$) planes in MnPS_3_,while 0.25 nm and 0.29 nm correspond to (131) and ($\bar{2}$01) planes in monoclinic unit cell of FePS_3_.

**Figure 3:**
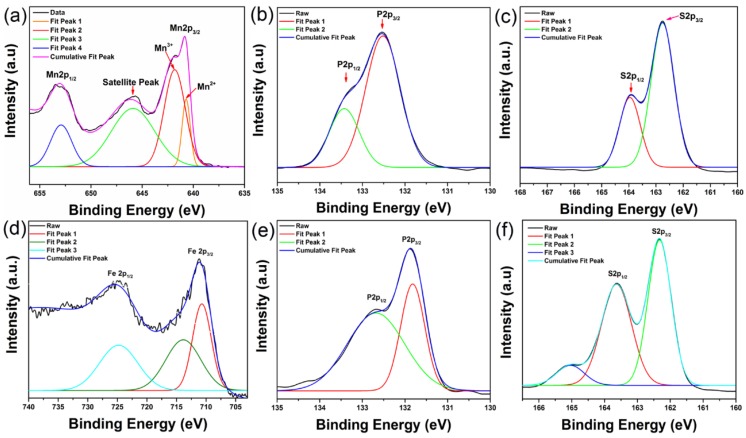
X-ray photoelectron microscopy of (**a**–**c**) MnPS_3_ and (**d**–**f**) FePS_3_ single crystals.

The chemical composition and bonding state of the samples were studied by using XPS analysis, as shown in Figure 3. The Mn 2p peaks of MnPS_3_ detected at 652.8 and 641 eV can be assigned to 2p_3/2_ and 2p_1/2_, respectively. The S 2p, peaks identified at 162.75 and 163.9 eV can be assigned to 2p_3/2_ and 2p_1/2_. For P 2p, two peaks at 132.5 eV and 133.6 eV are assigned to 2p_3/2_ and 2p_1/2_. In case of FePS_3_, the Fe 2p peaks at 709 and 722 eV are assigned to 2p_3/2_ and 2p_1/2_. Both S 2p and P 2p peaks of FePS_3_ show quite similar position and shape as those peaks for MnPS_3_. All these values are consistent with the previous reports [26,27].

**Figure 4:**
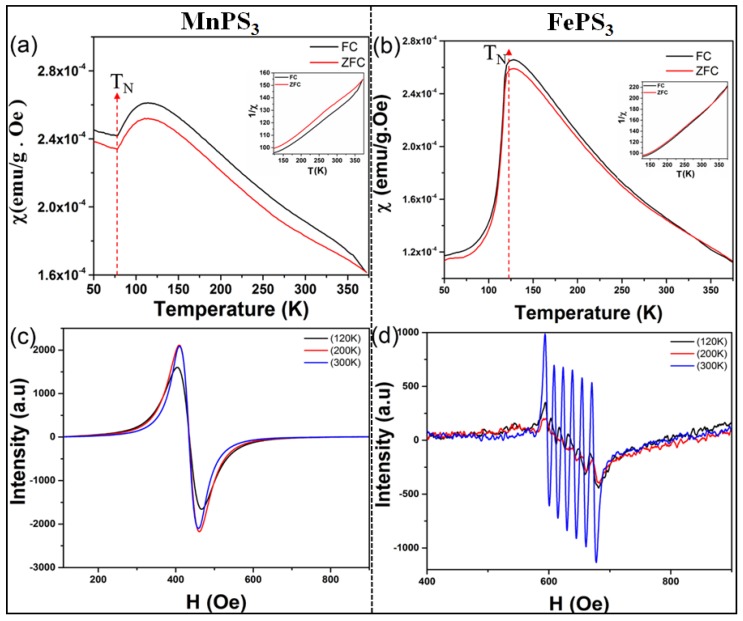
Magnetic susceptibility of (**a**) MnPS_3_ and (**b**) FePS_3_ single crystals with millimeter size. Electron paramagnetic resonance spectra of (**c**) bulk MnPS_3_ and (**d**) FePS_3_ single crystals.

Magnetic susceptibility indicates the magnetic properties of a material. It depicts the degree of magnetization of a material with respect to temperature at constant applied magnetic field. The magnetic susceptibility (χ) as a function of temperature for both MnPS_3_ and FePS_3_ crystals is represented in Figure 4a,b. The susceptibility plots of MnPS_3_ and FePS_3_ were examined through field cooling (FC) and zero field cooling (ZFC) curves. From the susceptibility plots, we can observe the isotropic behavior of MnPS_3_, while for FePS_3_ a considerable anisotropic trend has been noticed. The susceptibilities of MnPS_3_ and FePS_3_ increase with decreasing temperature till at 120 K and 128 K, respectively. Beyond this point it shows rapid decrease, exhibiting a Curie like tail at lowest temperatures. Furthermore, the susceptibility is seen to follow the Curie-Weiss law at higher temperatures (inset Figure 4a,b), which is an identification feature of the antiferromagnetic nature of these materials [7]. It can be inferred from Figure 4a,b that Neel temperature for MnPS_3_ and FePS_3_ are 78 K and 123 K, while the corresponding Curie temperatures are found to be at 365 K and 324 K, respectively. These values of Neel temperature and Curie temperature are well matched with the previous reports [28,29]. The broad maxima above Neel temperature is a result of short-range spin-spin correlation in typical magnetic systems. We can observe that magnetic susceptibility for FC is higher than ZFC throughout the observed range for both MnPS_3_ and FePS_3_. The increased Curie temperature beyond room temperature shows that these materials are favorable for spintronic applications.

The electron paramagnetic resonance (EPR) method was employed to study the quantum mechanical mixed spin states of both samples. The EPR signal typically originates from the surface of electrons, which increases by the exposures of some metals. The EPR spectra of the samples were measured at 120 K, 200 K and 300 K at constant frequency of 9 GHz. Figure 4c,d depicts the EPR spectra of MnPS_3_ and FePS_3_ with unpaired electrons. For MnPS_3_, the EPR spectra is isotropic, with the magnetic signals containing a single narrow resonance peak (Figure 4c). On the other hand, EPR spectra for FePS_3_ is highly anisotropic with multiple magnetic resonance peaks (Figure 4d). The magnetic resonance peaks for MnPS_3_ occurs at 404.1, 410 and 410.33 mT, while multiple resonance peaks were observed for FePS_3_ at all measured temperatures as described in Table 1. The strong signals at 300K for FePS_3_ can be attributed to the shortening of relaxation time with temperature in anisotropic materials [30,31]. Details for the position of six resonance peaks observed for FePS_3_ along with their corresponding values of g-factor is given in Table 1.

The magnetic coupling factor or g-factor “g_eff_” was computed from EPR spectra using the following relationship [32]:

(1) $$g_{\mathrm{eff}}≅\frac{h\gamma }{\mu_{B}H_{\mathrm{center}}}$$

where “γ” is the frequency, “h” is Planck constant, “μ_B_” indicating Bohr magnetron and H_center_ is the resonance magnetic field. The calculated values of g_eff_ are found to be 1.5921, 1.5684, and 1.5679 at 120, 200 and 300 K for MnPS_3_.

Anisotropy of magnetization originates from the anisotropy of the particles themselves in the form of shape or crystalline anisotropy and degree of alignment. The crystalline anisotropy relies on lattice forces and subsequent magnetization along an easy axis. EPR spectrum intensity is affected by number, position and nature of magnetic ions as well as the spin environment e.g., electron spin exchange with identical and non-identical atoms or molecules or with the spin of unpaired electrons of neighboring molecules. Additionally, in case of single crystals having equal orientations, it is expected that one defect will exist in all orientations with equal probability. For a low symmetry g-tensor and at an arbitrary direction of magnetic field, different effective values of g-factors are possible for the different orientations of the defect that can give rise to different magnetic field positions of EPR lines [33]. In fact, isotropy and anisotropy also affect the dielectric and absorption properties of materials. Isotropic materials display uniform dielectric functions along different directions, whereas anisotropic materials possess direction-dependent dielectric properties. In order to further understand the effect of isotropic and anisotropic behavior, spin-polarized first-principle density functional theory (DFT) calculations were performed to describe the magnetic response of MPS_3_ (M = Fe, Mn). The computations were carried out by using DFT through Vienna ab initio simulation package (VASP) [34]. A projector augmented wave (PAW) method [35,36] was used to characterize the ion-electron interface and the generalized gradient approximation (GGA) was stated by the PBE functional [37,38]. The plane wave cutoff energy was set to 500 eV with energy precision of 10^−5^ eV. The force criteria on each atom was less than 10^−2^ eV/Å. The Brillouin zone Γ-centered was sampled with a 12 × 12 × 1 Monkhorst−Pack of k-points grid for geometry optimization, while the static electronic calculation 8 × 8 × 1 and self-consistent calculations of the MPS_3_ (M = Mn, Fe) system were made. Unit cells were considered for these calculations. To obtain electronic and magnetic results, we employed a partition by hybrid HSE06 functional [38,39] with an accurate Fock exchange and typically achieved much better results than the DFT and DFT+U methods [40]. A single unit cell with lattice parameters of MnPS_3_ (a = 6.076 Å, b = 10.524 Å and c = 6.796 Å) and FePS_3_ (a = 5.949 Å, b = 10.288 Å and c = 6.72 Å) from the experimental data has been used for calculations. 

According to our magnetic energy calculations that are listed in Table 2, the ferromagnetic state in both cases is less stable as compared to the antiferromagnetic state, with an energy difference of about 0.601 eV for MnPS_3_, while for FePS_3_ the difference in energies is around 1.02 eV. Total energy calculations clearly defined that the room temperature antiferromagnetic (RTAFM) state is in the ground state of MPS_3_ (M = Fe, Mn).

**Figure 5:**
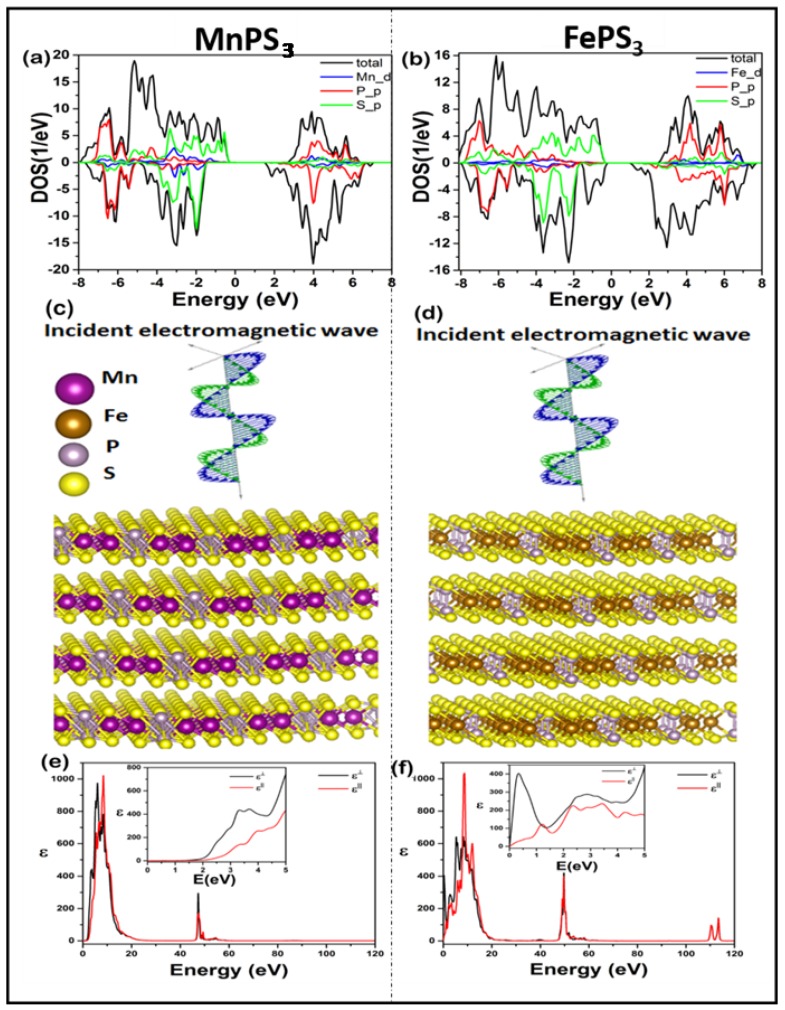
(**a**,**b**) Projected density of states of MnPS_3_ and FePS_3_ (**c**,**d**) structure of MnPS_3_ and FePS_3_ under incident electromagnetic wave (**e**,**f**) calculated dielectric function for MnPS_3_ and FePS_3_, respectively.

Similarly, the density of states (DOS) calculations in Figure 5 show the asymmetry between the spin-up and spin-down state of MPS_3_ (M = Fe, Mn). Figure 5a,b shows the evidence of magnetism for both MnPS_3_ and FePS_3_ layers with asymmetric density states in different energy levels. It can be seen that the electronic density of MnPS_3_ mainly stems from M’*d*, P’*p* and S’*p* states. Particularly, strong hybridization of Mn’*d*, P’*p* and S’*p* states is found between −7 eV and −1 eV, while strong P’*p* and weak Mn’*d*, and S’*p* was witnessed between 4 eV and 5 eV. However, near the Fermi level, strong S’*p* and weak Mn’*d* states exist, as shown in Figure 5a. Similarly for FePS_3_, Fe’*d*, P’*p* and S’*p* states exist between −7 eV and −2 eV, and strong P’*p* and weak Mn’*d*, and S’*p* appear between 4 eV and 6 eV. Likewise, strong S’*p* and a weak Fe’*d* states exist near the Fermi level, as shown in Figure 5b. All these states (spin-up and spin-down) are asymmetric and contribute to the magnetic isotropic/anisotropic trend throughout the energy range. The *d*-orbitals of Mn and Fe have the most obvious contribution of spinpolarization, which mainly contributes towards the magnetism of MPS_3_.

The frequency-dependent dielectric functions have been calculated using parallel and perpendicular electric field component vectors along the c-axis, as shown in Figure 5c,d. The frequency-dependent dielectric functions in Figure 5e,f show a strong isotropic and anisotropic behavior in lower and higher energy ranges for MnPS_3_ and FePS_3_, respectively. It was found that MnPS_3_ shows strong isotropic behavior for perpendicular and parallel polarization at lower and higher energy ranges. On the contrary, FePS_3_ exhibits a strong anisotropic trend for both perpendicular and parallel polarization with an increased number of peaks for dielectric constants at the given energy range. Moreover, the calculated frequency-dependent dielectric functions show a similar trend between 5 eV to 40 eV and 60 eV to 100 eV with increased number peaks of FePS_3_ for both perpendicular and parallel polarization as compared to MnPS_3_, which clearly demonstrates the effect of isotropic and anisotropic trend on MnPS_3_ and FePS_3_, respectively.

## 4. Conclusions

High-quality single crystals of MnPS_3_ and FePS_3_ were synthesized by using CVT. The EPR and VSM were used to corroborate the existence of antiferromagnetic behavior and to study isotropy/anisotropy in these materials. The probe for the magnetic character of MnPS_3_ and FePS_3_ was carried out at 120 K, 200 K and 300 K. The susceptibility showed a typical antiferromagnetic behavior in MnPS_3_ with an isotropic trend, while an anisotropic behavior was witnessed for FePS_3_. Moreover, our calculations further confirmed that the antiferromagnetic state was additionally stable than the ferromagnetic state for both MnPS_3_ and FePS_3_. The projected density of states (PDOS) manifested a vital irregularity between spin-up and spin-down channels of Mn and Fe 3d states, which is responsible for the primary contribution to the antiferromagnetic states near the Fermi level. The frequency-dependent dielectric function calculations have shown that MnPS_3_ possessed strong isotropic behavior for both perpendicular polarization and parallel polarization at lower and higher energy ranges. On the contrary, FePS_3_ exhibited strong anisotropic trend for both perpendicular as well as for parallel polarizations at the given energy range.

## Figures and Tables

**Table 1:** Resonance peak position and value of g-factor for bulk FePS_3_ single crystals.

Temperature	Value of *g*-Factor (Resonance Peak Position, mT)					
	Peak 1	Peak 2	Peak 3	Peak 4	Peak 5	Peak 6
300 K	1.080 (593.62)	1.057 (608.36)	1.032 (623.11)	1.007 (638.5)	0.983 (653.9)	0.9589 (670.6)
200 K	1.088 (590.9)	1.062 (605.0)	1.036 (621.0)	1.007 (638.5)	0.986 (651.85)	0.9592 (670.6)
120 K	1.082 (594.27)	1.066 (602.95)	1.043 (616.39)	1.018 (631.13)	0.990 (649.14)	0.9646 (666.59)

**Table 2:** Total energy calculations for ferromagnetic (FM) and anti-ferromagnetic (AFM) state and their difference for MnPS_3_ and FePS_3_.

Materials	E_0_ (FM Calculation)	E_0_ (AFM Calculation)	dE_0_ (FM-AFM)
MnPS_3_	114.92800 eV	114.32689 eV	0.601 eV
FePS_3_	164.99485 eV	163.97464 eV	1.02 eV
